# Growth differentiation factor 15 (GDF-15) is a potential biomarker of both diabetic kidney disease and future cardiovascular events in cohorts of individuals with type 2 diabetes: a proteomics approach

**DOI:** 10.1080/03009734.2019.1696430

**Published:** 2019-12-05

**Authors:** Axel C. Carlsson, Christoph Nowak, Lars Lind, Carl Johan Östgren, Fredrik H. Nyström, Johan Sundström, Juan Jesus Carrero, Ulf Riserus, Erik Ingelsson, Tove Fall, Johan Ärnlöv

**Affiliations:** a Department of Neurobiology, Care Sciences and Society (NVS), Karolinska Institutet, Huddinge, Sweden;; b Department of Medical Sciences, Uppsala University, Uppsala, Sweden;; c Department of Medical and Health Sciences, Linköping University, Linköping, Sweden;; d Department of Medical Epidemiology and Biostatistics, Karolinska Institutet, Stockholm, Sweden;; e Department of Public Health and Caring Sciences, Clinical Nutrition and Metabolism, Uppsala University, Uppsala, Sweden;; f Department of Medicine, Division of Cardiovascular Medicine, Stanford University School of Medicine, Stanford, CA, USA;; g Stanford Cardiovascular Institute, Stanford University, Stanford, CA, USA;; h Stanford Diabetes Research Center, Stanford University, Stanford, CA, USA;; i Molecular Epidemiology and Science for Life Laboratory, Uppsala University, Uppsala, Sweden;; j School of Health and Social Studies, Dalarna University, Falun, Sweden

**Keywords:** Albumin-creatinine ratio, biomarker, diabetic kidney disease, glomerular filtration rate, proteomics, risk factor, type 2 diabetes mellitus

## Abstract

**Background:** Diabetic kidney disease (DKD) is a leading risk factor for end-stage renal disease and is one of the most important risk factors for cardiovascular disease in patients with diabetes. It is possible that novel markers portraying the pathophysiological underpinning processes may be useful.

**Aim:** To investigate the associations between 80 circulating proteins, measured by a proximity extension assay, and prevalent DKD and major adverse cardiovascular events (MACE) in type 2 diabetes.

**Methods:** We randomly divided individuals with type 2 diabetes from three cohorts into a two-thirds discovery and one-third replication set (total *n* = 813, of whom 231 had DKD defined by estimated glomerular filtration rate <60 mg/mL/1.73 m^2^ and/or urinary albumin-creatinine ratio ≥3 g/mol). Proteins associated with DKD were also assessed as predictors for incident major adverse cardiovascular events (MACE) in persons with DKD at baseline.

**Results:** Four proteins were positively associated with DKD in models adjusted for age, sex, cardiovascular risk factors, glucose control, and diabetes medication: kidney injury molecule-1 (KIM-1, odds ratio [OR] per standard deviation increment, 1.65, 95% confidence interval [CI] 1.27–2.14); growth differentiation factor 15 (GDF-15, OR 1.40, 95% CI 1.16–1.69); myoglobin (OR 1.57, 95% CI 1.30–1.91), and matrix metalloproteinase 10 (MMP-10, OR 1.43, 95% CI 1.17–1.74). In patients with DKD, GDF-15 was significantly associated with increased risk of MACE after adjustments for baseline age, sex, microalbuminuria, and kidney function and (59 MACE events during 7 years follow-up, hazard ratio per standard deviation increase 1.43 [95% CI 1.03–1.98]) but not after further adjustments for cardiovascular risk factors.

**Conclusion:** Our proteomics approach confirms and extends previous associations of higher circulating levels of GDF-15 with both micro- and macrovascular disease in patients with type 2 diabetes. Our data encourage additional studies evaluating the clinical utility of our findings.

## Introduction

Diabetic kidney disease (DKD) contributes to up to half of all cases of end-stage renal disease in the world and is one of the most important risk factors for cardiovascular disease in patients with diabetes (1). The definition of DKD relies on assessment of both kidney function and kidney damage and is defined as an estimated glomerular filtration rate (eGFR) of less than 60 mg/mL/1.73 m^2^ and/or micro- or macroalbuminuria in patients with diabetes (1).

Recent technological advances have made it possible to simultaneously measure a large number of proteins in biological samples (2,3). These ‘proteomics’ assays could offer new ways to discover pathophysiologic pathways and identification of novel disease biomarkers in DKD. Yet, despite the substantial clinical relevance of DKD as one of the most common complications of both types of diabetes, only a few prior proteomics studies have focussed on DKD (4,5), and in most of these prior studies, the definition of DKD did not include albuminuria assessments (4). We believe that proteomics analyses can provide novel insights into underlying mechanisms leading to DKD but also to mechanisms that mediate the risk of future cardiovascular disease. Estimated GFR and albuminuria are well-established biomarkers of kidney disease progression. However, much is still unknown about the pathophysiology of cardiovascular disease-specific to DKD. We reasoned that associations between circulating proteins previously linked to cardiovascular disease (CVD) and inflammation could provide new insights into cardiovascular disease pathways involved in DKD. The kidney is one of the best-perfused organs in the body and shares many biomarkers that are also of interest for cardiovascular pathology.

Therefore, we aimed to explore and validate the associations between 80 circulating proteins involved in cardiovascular pathology or inflammation with DKD in persons with type 2 diabetes enrolled in three separate cohort studies. We also aimed to study if the identified proteins were associated with the incidence of major adverse cardiovascular events beyond established risk factors in those with prevalent DKD.

## Methods

### Study cohorts

Data were used from individuals with type 2 diabetes enrolled in three cohorts where eGFR and microalbuminuria were measured on at least one occasion, and a biobank with samples available for proteomic analysis: the Cardiovascular Risk Factors in Patients with Diabetes: a Prospective Study in Primary Care (CARDIPP) (6), the Prospective Investigation of the Vasculature in Uppsala Seniors (PIVUS) (7), and Uppsala Longitudinal Study of Adult Men (ULSAM) (8).

The CARDIPP study was launched in 2005, and the baseline data collection was completed in November 2008. Patients with type 2 diabetes aged 55–65 were consecutively recruited during their usual annual follow-up assessments at 22 primary healthcare diabetes clinics in the Swedish counties of Östergötland and Jönköping (9). The centres varied in size and were located in different sociodemographic areas, but all followed the national guidelines for diabetes care. Out of 761 consecutively enrolled patients, 621 with available data on proteomics, cardiovascular risk factors, DKD status, and outcome data on cardiovascular events were included in the present analyses.

All 70-year-old men and women living in Uppsala, Sweden, between 2001 and 2004 were invited to participate in the PIVUS study (http://www.medsci.uu.se/pivus/pivus.htm) (6) and were re-investigated with blood samples and urine biochemistry at the age of 75 years. At the re-investigation, 77 participants had diabetes and were thus included in this study.

The ULSAM study was initiated in 1970 (7). All 50-year-old male residents of Uppsala, Sweden, who had been born in 1920–24 were invited to participate in a health survey of cardiovascular risk factors (described in detail here: http://www.pubcare.uu.se/ULSAM) (8). At the fourth examination cycle, when participants were approximately 77 years old, 1398 were invited and 838 (60%) participated, of which 115 persons had diabetes and could be included in the present study.

### Outcome definitions, inclusion criteria, and number of eligible participants

We defined type 2 diabetes as fulfilling at least one of the following criteria: (i) self-reported type 2 diabetes; (ii) physician-diagnosed type 2 diabetes according to hospital records; (iii) fasting glucose ≥7.0 mmol/L (126 mg/dL); or iv) HbA1c >6.5% (48 mmol/mol). Participants without available frozen plasma or serum samples or with missing data on proteomics, eGFR, or microalbuminuria were excluded. DKD was defined as an eGFR below 60 mg/mL/1.73 m^2^ and/or urinary albumin-creatinine ratio (ACR) ≥3 g/mol. Single measurements of eGFR and ACR were assessed.

Major adverse cardiovascular events (MACE) were defined as fatal or non-fatal myocardial infarction (International Classification of Diseases, 10th ed., I21) or stroke (I60–I63), whichever occurred first after baseline assessment. These were obtained from follow-up in national Swedish registers that started after the baseline investigation in each individual.

By combining data from the three cohorts, the total study population was 813, of whom 231 had prevalent DKD. There were 59 MACE recorded after baseline in those with DKD.

### Ethical permission

Participants provided written informed consent, and the study was conducted according to the Declaration of Helsinki. Ethical permission was granted by the ethics committees of Linköping University and Uppsala University.

### Multiplex protein assay

The Olink Proseek Multiplex Cardiovascular I 96 x 96 kit was used to measure proteins in plasma (CARDIPP, PIVUS) and serum (ULSAM) by real-time polymerase chain reaction (PCR) using the Fluidigm BioMark HD real-time PCR platform. The assay attempts to quantify the abundance of 92 proteins and uses the standard 96-wells plate format. Of the 96-wells, one serves as negative control, whilst three wells contain positive controls. The resulting relative values obtained were log_2_-transformed for subsequent analysis. Twelve proteins with <85% valid measurements were removed, leaving 80 proteins for the present analysis. If values were below the lower limit of detection (LOD), they were imputed by LOD/2. Each protein was normalized by plate (by setting the mean = 0, and standard deviation = 1 within each plate) and by storage time (correction based on the observed values and predicted values from a spline model). In a previous validation study of the proteomics assay, the mean intra-assay coefficient of variation was found to be 8%, and the mean inter-assay coefficient of variation was 12% (10). Detailed information about the methods used in the assay and on the coefficients of variation of specific proteins can be found on the Olink website (www.Olink.com).

### Statistical analysis

We used mixed-effects logistic regression to assess associations between protein abundance (standardized to a mean of 0 and a standard deviation of 1) and DKD, with adjustments for age, sex (fixed effects), and cohort (random effect). Data were divided into a discovery data set and a replication data set. Samples were combined at the individual person-level and randomly split into a two-thirds training and one-third hold-out test set using the ‘createDataPartition’ function in the ‘caret’ package in R. The function balances the DKD case proportion across both samples. Proteins associated at a 5% false discovery rate (FDR) in the discovery sample were tested in the replication sample and were considered successfully replicated at the nominal significance level of 0.05 (11). Missing covariate values were imputed by multivariate imputation by chained equations (MICE) by predictive mean matching based on all other covariates and averaged across five iterations (12). Imputed values were compared against complete values to assess accuracy.

As a second step, we used the whole cohort to perform additional multivariable modelling adjusted for cohort (random effects), age, sex, glucose control/diabetes factors (HbA1c, oral antidiabetic drug use, and insulin treatment), and cardiovascular risk factors (cardiovascular disease at baseline, low-and high-density lipoprotein cholesterol, triglycerides, BMI, cardiovascular disease at baseline, systolic and diastolic blood pressure, antihypertensive therapy, statins, and smoking status).

Finally, we used Cox regression with frailty effect for cohort adjusted for age, sex, GFR, and microalbuminuria to study if any of the proteins associated with DKD were associated with risk of MACE beyond baseline ACR and eGFR in individuals with DKD (13). We also adjusted a model additionally for cardiovascular risk factors (prevalent cardiovascular disease at baseline, systolic blood pressure, low-density lipoprotein cholesterol, and smoking). All statistical analyses were performed with R version 3.3.2, 2016–10-31 (14).

## Results

### Baseline characteristics

A total of 813 subjects were included in the present study, of whom 231 had prevalent DKD. The sample was divided into discovery 542 (two-thirds) and replication 271 (one-third). Baseline characteristics are shown in Table 1 for the whole cohort and also stratified by DKD status. Microalbuminuria was more common than an eGFR <60 mg/mL/1.73 m^2^ among those with DKD, 71% versus 39%. Systolic blood pressure was higher in those with DKD (147 mmHg) versus those without DKD (139 mmHg), and previous cardiovascular disease was also more common, 44% versus 25%.

**Table 1.** Baseline characteristics.

Variables	All (*n* = 813)	DKD (*n* = 231)	No DKD (*n* = 582)
Age, y	64 ± 7	68 ± 8	63 ± 6
Women	234 (29%)	62 (27%)	174 (30%)
Glomerular filtration rate (eGFR, mL/min)	77 ± 14	70 ± 17	80 ± 12
Glomerular filtration rate (eGFR, mL/min) <60	90 (11%)	90 (39%)	0
Microalbuminuria (albumin-creatinine ratio ≥3 g/mol)	164 (20%)	164 (71%)	0
Body mass index (BMI, kg/m^2^)	30 ± 4.6	30 ± 4.5	30 ± 4.6
Systolic blood pressure (mmHg)	142 ± 20	147 ± 19	139 ± 20
Diastolic blood pressure (mmHg)	81 ± 11	82 ± 10	81 ± 11
Fasting glucose (mmol/L)	8.7 ± 2.6	8.9 ± 3.0	8.6 ± 2.4
Triglycerides (mmol/L)	1.8 ± 1.1	1.9 ± 1.1	1.8 ± 1.1
Low-density lipoprotein cholesterol (mmol/L)	2.7 ± 0.8	2.7 ± 0.8	2.8 ± 0.8
High-density lipoprotein cholesterol (mmol/L)	1.3 ± 0.3	1.2 ± 0.3	1.3 ± 0.3
Glycated haemoglobin (HbA1c, mmol/L)	51 ± 12	53 ± 13	51 ± 12
Treatment with insulin	209 (26%)	66 (29%)	143 (25%)
Oral antidiabetic drug treatment	480 (59%)	152 (66%)	328 (56%)
Previous cardiovascular disease	247 (30%)	101 (44%)	146 (25%)
Statin treatment	415 (51%)	125 (54%)	290 (50%)
Smoking	123 (15%)	28 (12%)	95 (16%)

Data are shown as mean ± SD, or as *n* (%).

### Associations between proteins and prevalent DKD

**Figure 1.**
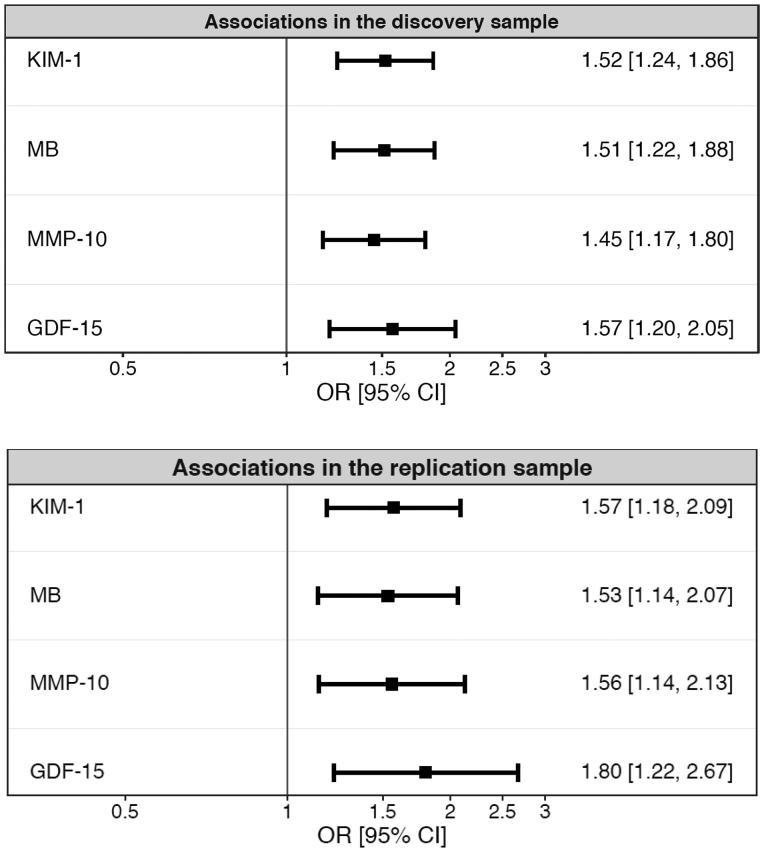
Discovery and replication of the association between 80 circulating proteins measured by a proximity extension assay, and prevalent diabetic kidney disease. Proteins associated at a 5% false discovery rate in the discovery sample were tested in the replication sample, and considered successfully replicated at the nominal significance level of 0.05. Two-thirds of the subjects (*n* = 542) were analyzed in the discovery sample, and one-third of the subjects (*n* = 271) were analyzed in the replication sample.

A total of 14 proteins were positively associated with DKD in the discovery sample at <5% FDR in age-, sex-, and cohort-adjusted models. Four of these 14 proteins were associated with DKD in the replication sample: kidney injury molecule-1 (KIM-1), growth differentiation factor 15 (GDF-15), myoglobin, and matrix metalloproteinase 10 (MMP-10) (Figure 1). Higher levels of all four proteins remained significantly associated with prevalent DKD in additional multivariable models that were adjusted for cardiovascular risk factors, glucose control, and treatment for type 2 diabetes (Table 2).

**Table 2.** Multivariable logistic regression models for the association between discovered and replicated proteins and diabetic kidney disease in the whole sample.

Protein	Model A	Model B	Model C
KIM-1			
OR (95% CI)	1.67 (1.31–2.13)	1.59 (1.25–2.03)	1.61 (1.24–2.09)
* p* value	3.23 × 10^−5^	1.6 × 10^−4^	3.18 × 10^−4^
GDF-15			
OR (95% CI)	1.47 (1.22–1.77)	1.43 (1.20–1.70)	1.38 (1.14–1.67)
* p* value	3.66 × 10^−5^	5.6 × 10^−5^	7.34 × 10^−4^
MB			
OR (95% CI)	1.51 (1.25–1.83)	1.56 (1.30–1.88)	1.55 (1.28–1.89)
* p* value	1.83 × 10^−5^	2.9 × 10^−6^	8.29 × 10^−6^
MMP-10			
OR (95% CI)	1.46 (1.20–1.78)	1.42 (1.19–1.70)	1.42 (1.67–1.73)
* p* value	1.32 × 10^−4^	1.2 × 10^−4^	4.82 × 10^−4^

The following was adjusted for in the logistic regression models: Model A: age, sex, cardiovascular risk factors and cohort; Model B: age, sex, glucose control/diabetes factors, and cohort; Model C: all relevant factors in model C ([A and B combined], HbA1c, oral antidiabetic drug use and insulin treatment, low- and high-density lipoprotein, triglycerides, body mass index, cardiovascular disease at baseline, systolic and diastolic blood pressure, antihypertensive therapy, statins, and smoking status).

GDF-15: growth differentiation factor 15; KIM-1: kidney injury molecule 1; MB: myoglobin; MMP-10: matrix metalloproteinase 10.

### Associations of proteins and MACE incidence in individuals with prevalent DKD

Over a median of 7.9 ± 1.5 years of follow-up, 59 persons out of the 231 with DKD at baseline experienced a MACE event. In these, higher levels of GDF-15 at baseline were associated with a higher risk of incident MACE after additional adjustment for eGFR and ACR, while neither KIM-1, myoglobin, nor MMP-10 was associated with future MACE (Table 3). When we adjusted for cardiovascular risk factors the results were attenuated and not statistically significant.

**Table 3.** Cox regression models for the association between discovered and replicated circulating proteins and time to major adverse cardiovascular events.

Protein	Model A: Hazard ratio (95% CI)	*p* value	Model B: Hazard ratio (95% CI)	*p* value
KIM-1	1.15 (0.88–1.50)	0.31	1.07 (0.79–1.45)	0.66
GDF-15	1.43 (1.03–1.98)	0.03	1.34 (0.96–1.88)	0.09
MB	1.17 (0.88–1.55)	0.30	1.12 (0.83–1.51)	0.45
MMP-10	1.21 (0.89–1.65)	0.23	1.27 (0.93–1.75)	0.13

Model A was adjusted for age, sex, frailty effect for cohort, microalbuminuria, and kidney function; Model B for all variables in Model A and cardiovascular disease at baseline, smoking, low-density lipoprotein, and systolic blood pressure.

CI: confidence interval; KIM-1: kidney injury molecule 1; GDF-15: growth differentiation factor 15; MB: myoglobin; MMP-10: matrix metalloproteinase 10.

## Discussion

### Main findings

In our cross-sectional analysis of individuals with type 2 diabetes from three different cohorts, a multiplex proteomics assay identified four circulating proteins associated with DKD: KIM-1, GDF-15, myoglobin, and MMP-10. The essentially unchanged associations of these biomarkers with DKD after adjustment for glycemic control, diabetes medication, and cardiovascular risk factors point to a possible independent prediction of these biomarkers beyond factors that are generally assessed in clinical practice. Moreover, in prospective analyses in participants with prevalent DKD in the present study, higher levels of GDF-15 were associated with a higher risk of incident MACE after adjustments for baseline eGFR and ACR. Additional adjustments for established cardiovascular risk factors attenuated this association slightly so that it was no longer statistically significant.

### Comparison with previous studies

Although the prevalence of DKD parallels the type 2 diabetes and obesity epidemic (15), there has been little advancement in the discovery of clinically relevant biomarkers for DKD. There are several examples of previous studies investigating individual proteins as biomarkers of DKD (4,5,16–18). However, we are aware of only few previous studies that have simultaneously evaluated multiple proteins as biomarkers of DKD progression in serum or plasma. In a recent report in patients with type 2 diabetes in Scotland, 205 circulating proteins were evaluated, of which 30 proteins (including GDF-15) were associated with rapid progression of eGFR decline (19). In another study in 82 patients with type 2 diabetes, a panel of 13 biomarkers representing fibrosis, angiogenesis, inflammation, mineral metabolism, and endothelial function was found to improve the prediction of eGFR decline (20). Few of the proteins evaluated in these two prior studies overlapped with the proteins evaluated in the present study. It should also be noted that, in these studies, albuminuria was not included as a kidney disease outcome. Moreover, none of these studies evaluated whether DKD-associated biomarkers predicted incident cardiovascular disease in those with prevalent DKD.

### GDF-15

GDF-15 is a cytokine-induced as a stress response in inflammatory states, after tissue injury and as a response to oxidative stress (21). A comprehensive research effort into GDF-15 (22), and its cardiometabolic associations, is currently ongoing by several research groups worldwide. GDF-15 is of interest in individuals with diabetes and has been shown to be a marker of elevated glucose during an oral glucose tolerance test and to be a marker of impaired fasting glucose, as well as a marker of metformin treatment (23–25). However, metformin treatment did not seem to affect the associations of GDF-15 with DKD in the present study, since diabetic treatment was adjusted for in our full model and as metformin is the most common oral antidiabetic drug in Sweden. Furthermore, reference intervals for GDF-15 have been suggested in DKD (26).

Higher levels of circulating GDF-15 have been linked to an increased risk for several adverse outcomes, including a recent study showing an association with incident type 2 diabetes (27), deteriorating microalbuminuria (28), progression of albuminuria in persons with type 2 diabetes (28), kidney function decline and cardiovascular risk in persons with type 1 diabetes (29), early death in patients undergoing haemodialysis (30), as well as incident heart failure and cardiovascular events in the general population (31). The fact that GDF-15 was the only biomarker that was associated with incident cardiovascular events is interesting and also supported by several studies showing associations between GDF-15 levels and both cardiovascular morbidity and mortality (32–35).

### KIM-1

KIM-1 is expressed in the proximal tubule and excreted in the urine, and urinary KIM-1 is used as a clinical marker of acute kidney damage (36–38). Less is known about plasma levels of KIM-1, but, out of 80 tested proteins, circulating KIM-1 was the biomarker that had the strongest association with ACR in the general population (3). Apart from the mechanisms of KIM-1 in acute kidney damage, experimental studies have shown that KIM-1 is active in the regulation of immune responses activated by the T helper cell (39). Circulating KIM-1 has been associated with the number of carotid arteries affected by atherosclerotic plaques in the general population (40), as well as coronary artery atherosclerosis, and the risk of cardiovascular death in dialysis patients (41). Whether circulating levels of KIM-1 reflect atherosclerosis in the kidney in these diabetes patients remains to be established.

### Myoglobin

Rhabdomyolysis is well known to be associated with acute kidney injury, and one of the proteins that are used as markers of rhabdomyolysis and its associated acute kidney injury is myoglobin (42). Although myoglobin has not been put forward as a DKD biomarker, plasma levels of myoglobin have been associated with chronic kidney disease, and higher levels of myoglobin with higher stages of chronic kidney disease (43). Our findings of an association between myoglobin and DKD suggest that myoglobin, in addition to its use in acute kidney injury, maybe a marker of slowly deteriorating kidney function in diabetes patients.

### MMP-10

Matrix metalloproteinases have been suggested to be causally involved in many processes leading to kidney disease progression and cardiovascular disease (44). Elevated levels of MMP-10 were independently associated with the severity of atherosclerosis in patients with chronic kidney disease (CKD) (45), and also associated with nephropathy in patients with type 1 diabetes (46). Interestingly, MMP-10 was not associated with eGFR-decline or ACR in previous community-based studies using the same assay (2,3) and may thus be DKD-specific. Matrix remodelling properties of MMP-10 and its degradation products favour expansion of a thin membrane supporting the capillary loops in renal glomeruli called the mesangium, which may explain some of its effects in the development of DKD (47). In fact, glucose-induced mesangial matrix remodelling has been suggested as a mechanism leading to nephropathy, and thus MMP-10 has been suggested as a potential drug target to slow down diabetic nephropathy and retinopathy (46).

### Strengths and limitations

Strengths of our investigation include the discovery/replication approach in multiple study samples, which add to the validity and generalizability of our findings. We cannot infer causality in the present study as it is of observational design. Limitations include a possible selection bias for persons participating in cohort studies that in general often are healthier than the average patient population. Another limitation is the fact that our study was based on single assessments of the proteins and kidney phenotypes. Limitations of the proteomics assay include that only relative levels of the proteins are obtained, which makes defining relevant cut-off limits impossible. Furthermore, the selection of the specific proteins on the Olink CVD-I assay was not based on potential relevance for DKD. Neither can we determine if it is the protein that has an effect on the kidney nor if it is the reduced clearance as an effect of reduced kidney function that explains our findings. Since we did not perform kidney biopsies in our study participants, we were not able to rule out the misclassification of DKD due to other causes. Finally, the limited sample size in our longitudinal analyses precluded stratified analyses in participants with versus without prevalent cardiovascular disease at baseline.

## Conclusions

We discovered and replicated four blood proteins associated with prevalent DKD. Circulating levels of GDF-15 were associated with incident cardiovascular events in models adjusted for age, sex, kidney function, and microalbuminuria; however, the association was attenuated when adjusted for established cardiovascular risk factors. Our study encourages more studies evaluating large-scale proteomics in order to discover new pathways leading to DKD and pinpoint prognostic markers of cardiovascular risk.
